# Foreign Body Granulomatous Reaction of the Breast Mimicking Carcinoma: A Radiologic-Pathologic Diagnostic Pitfall

**DOI:** 10.7759/cureus.106202

**Published:** 2026-03-31

**Authors:** Yanina Nikolaus

**Affiliations:** 1 Pathology, Marshall University Joan C. Edwards School of Medicine, Huntington, USA

**Keywords:** benign breast lesion, breast core needle biopsy, breast imaging mimic, foreign body granuloma, radiologic-pathologic correlation

## Abstract

Foreign body granulomatous reactions of the breast are uncommon benign entities that may closely mimic carcinoma on imaging. We report the case of a woman in her seventies who presented with persistent breast pain and an enlarging lesion detected on imaging. Mammography demonstrated architectural distortion and focal asymmetry, while ultrasound revealed an irregular hypoechoic mass with posterior acoustic shadowing, leading to classification as a Breast Imaging-Reporting and Data System (BI-RADS) 4 suspicious lesion. Ultrasound-guided core needle biopsy showed fibrous breast tissue with foreign body granulomatous inflammation and vacuolated spaces consistent with exogenous material, with no evidence of malignancy. Radiologic-pathologic correlation confirmed concordant benign findings, and surgical excision was avoided. The patient was managed conservatively with imaging surveillance and remained clinically stable on follow-up. This case highlights an important diagnostic pitfall in breast imaging and emphasizes the role of multidisciplinary radiologic-pathologic concordance in guiding appropriate management and preventing unnecessary surgical intervention.

## Introduction

Breast lesions demonstrating architectural distortion or irregular masses on imaging are often highly suspicious for malignancy and typically prompt tissue sampling. However, several benign processes may mimic carcinoma radiologically, including fat necrosis, post-procedural changes, and foreign body reactions. Recognition of these entities is important to avoid unnecessary surgical intervention [1-3].

Foreign body granulomatous reactions of the breast may occur following prior surgery, trauma, or exposure to exogenous materials. These reactions are characterized histologically by multinucleated giant cells and granulomatous inflammation surrounding vacuolated spaces representing dissolved foreign material [3]. Although uncommon, such lesions may present as irregular masses with posterior acoustic shadowing on ultrasound or architectural distortion on mammography, closely simulating invasive carcinoma [1,2]. This case highlights a foreign body granulomatous reaction presenting as a radiologically suspicious breast mass and emphasizes the importance of careful radiologic-pathologic correlation in establishing concordance and preventing overtreatment.

## Case presentation

A woman in her seventies presented for evaluation of an abnormal left breast imaging finding. She reported persistent left breast pain since undergoing a stereotactic biopsy at an outside institution approximately one year earlier. She denied a palpable mass, nipple discharge, skin changes, or nipple inversion. On physical examination, no palpable breast mass, skin abnormalities, or nipple changes were identified.

Her past breast history was notable for the removal of three benign breast lesions in the 1970s. She denied a history of breast implants but was uncertain whether any injectable materials or non-absorbable surgical materials had been used during prior procedures. The patient was referred for further evaluation after imaging demonstrated an enlarging abnormality within the left breast.

**Figure 1:**
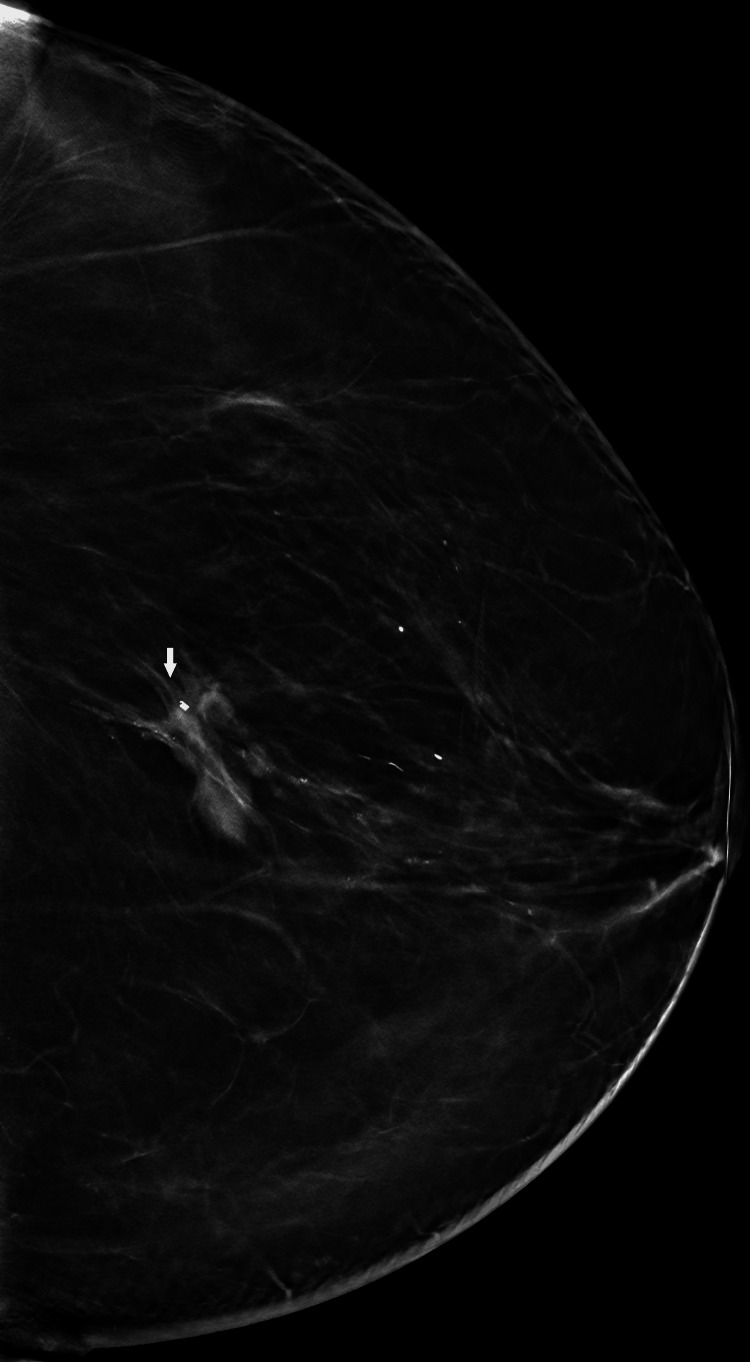
Diagnostic mammography demonstrating architectural distortion and focal asymmetry in the posterior left breast at approximately the 6 o’clock position, corresponding to a BI-RADS 4 lesion (arrow). BI-RADS: Breast Imaging-Reporting and Data System

**Figure 2:**
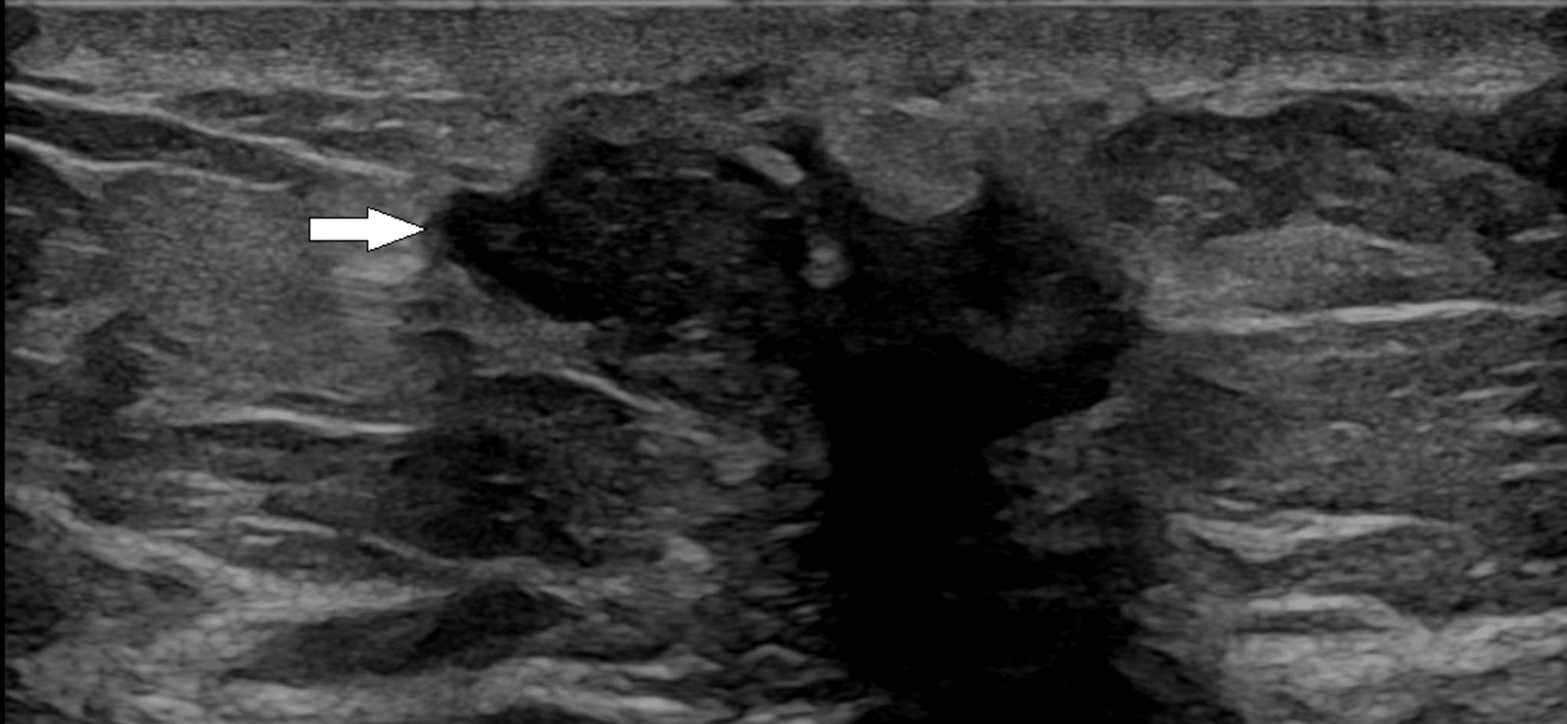
Targeted ultrasound demonstrating an irregular hypoechoic mass with posterior acoustic shadowing corresponding to the mammographic abnormality (arrow).

Diagnostic mammography demonstrated scattered fibroglandular density and an irregular focus of tissue asymmetry within the posterior left breast at the approximate 6 o’clock position, measuring approximately 2.2 cm. The lesion demonstrated architectural distortion and was classified as BI-RADS category 4 (Figure 1). Targeted ultrasound revealed a corresponding irregular hypoechoic mass with posterior acoustic shadowing (Figure 2).

**Figure 3:**
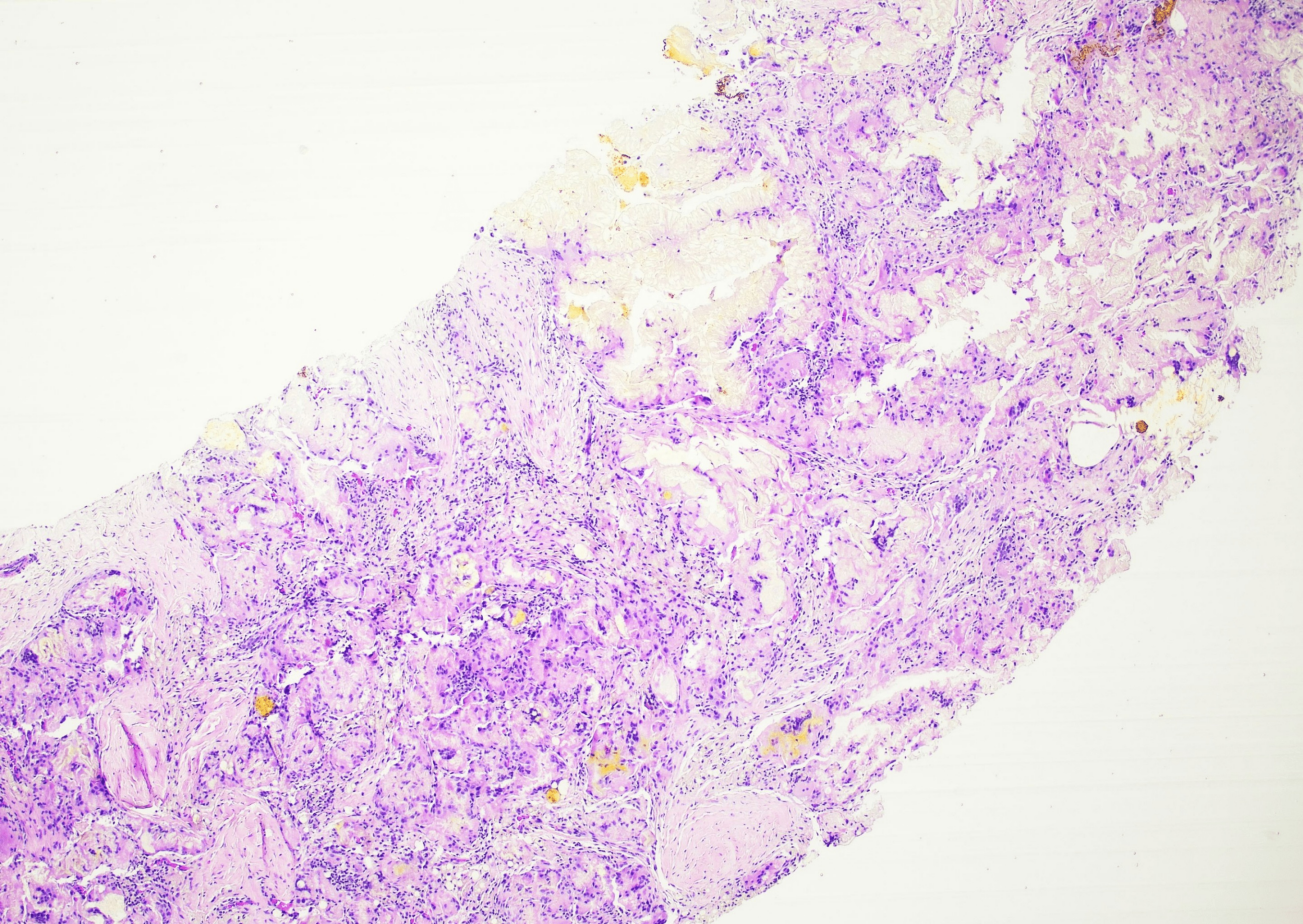
Core needle biopsy showing fibrous breast tissue with foreign body granulomatous inflammation composed of multinucleated giant cells and stromal reaction (H&E, ×4).

**Figure 4:**
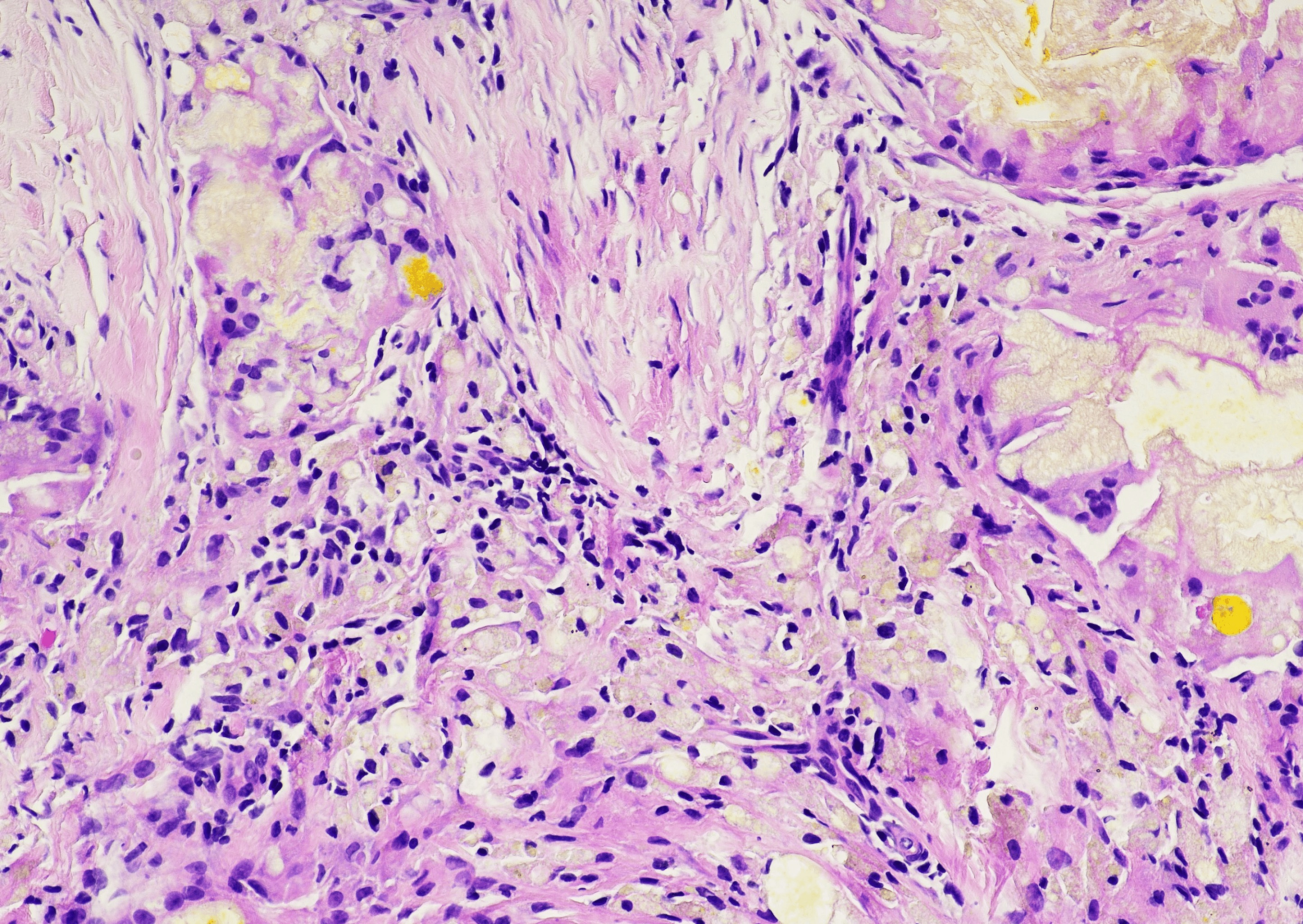
Higher magnification showing multinucleated foreign body-type giant cells surrounding vacuolated spaces consistent with dissolved foreign material (H&E, ×20).

Ultrasound-guided core needle biopsy was performed. Histologic examination showed fibrous breast tissue with multinucleated giant cells and granulomatous inflammation surrounding vacuolated spaces consistent with foreign body granulomatous reaction (Figure 3). At higher magnification, multinucleated foreign body-type giant cells were seen surrounding vacuolated spaces compatible with dissolved exogenous material (Figure 4). No epithelial atypia or malignancy was identified.

Based on imaging findings, the primary diagnostic consideration was invasive breast carcinoma. Additional considerations included fat necrosis and post-procedural scarring, given the patient’s history of prior breast procedures.

Because histopathologic findings were benign and radiologic-pathologic correlation was deemed concordant, surgical excision was not recommended. The patient was managed conservatively with imaging surveillance.

At follow-up evaluation, the patient remained clinically stable without interval progression of imaging findings. Continued imaging surveillance was recommended.

## Discussion

Foreign body granulomatous reactions of the breast are uncommon benign processes that may develop following prior surgery, trauma, or exposure to exogenous materials, including silicone, suture material, or other injected substances. Histologically, these reactions are characterized by multinucleated foreign body-type giant cells and granulomatous inflammation surrounding vacuolated spaces representing dissolved foreign material [3].

Radiologically, these lesions may present as irregular masses, architectural distortion, or areas of focal asymmetry on mammography and may demonstrate posterior acoustic shadowing on ultrasound. These imaging features overlap substantially with those seen in invasive breast carcinoma and frequently lead to classification as suspicious lesions requiring biopsy [1,2]. Benign processes such as fat necrosis, post-procedural changes, and foreign body reactions are well-recognized mimics of malignancy on breast imaging [1,2].

Several reports have described foreign body granulomatous reactions of the breast presenting as suspicious lesions on imaging and mimicking invasive carcinoma [1-3]. Selected previously reported cases illustrating this diagnostic pitfall are summarized in Table 1. These cases demonstrate that such lesions often present as radiologically suspicious abnormalities and require tissue sampling with careful radiologic-pathologic correlation to establish a benign diagnosis and guide appropriate management. Similar to previously reported cases, the present case highlights that foreign body granulomatous reactions may closely mimic invasive carcinoma on imaging but can be accurately diagnosed on core biopsy when radiologic-pathologic concordance is appropriately assessed.

**Table 1:** Selected reports describing fat necrosis and foreign body granulomatous reactions of the breast presenting as radiologically suspicious lesions mimicking malignancy and their management. BI-RADS: Breast Imaging-Reporting and Data System

Study	Patient Age	Etiology/Trigger	Imaging Appearance	Pathologic Findings	Diagnostic Challenge	Management
Bilgen et al. [1]	Middle-aged to elderly women	Prior surgery or trauma	Architectural distortion, spiculated mass, or calcifications	Fat necrosis with granulomatous inflammation	Imaging findings mimicking carcinoma	Conservative management or biopsy for diagnosis
Kerridge et al. [2]	Variable	Surgery, trauma, radiation	Irregular mass, hypoechoic lesion, posterior shadowing	Fat necrosis with histiocytic and granulomatous reaction	Radiologic–pathologic correlation required	Core needle biopsy
Yazici et al. [3]	Adult	Foreign body reaction to surgical material	Suspicious breast mass	Foreign body granuloma with multinucleated giant cells	Lesion mimicked carcinoma clinically and radiologically	Surgical excision
Liberman [4]	Adult	Suspicious breast lesion	BI-RADS suspicious abnormality	Benign pathology on core biopsy	Imaging–pathology concordance assessment	Imaging surveillance if concordant
Bruening et al. [5]	Adult	Suspicious breast lesion	Imaging abnormality	Benign or malignant depending on biopsy	Choice of biopsy method	Core needle biopsy vs surgical biopsy
Present case	70s	Remote breast surgery	BI-RADS 4 mass with distortion and shadowing	Foreign body granulomatous reaction	Suspicious imaging resolved by concordant pathology	Imaging surveillance

Foreign body reactions may occasionally present years or even decades after the inciting exposure, particularly when non-absorbable materials or injected substances were used during prior procedures [3]. These delayed presentations may create diagnostic uncertainty, especially when patients have remote surgical histories or limited procedural documentation. In the present case, the patient’s prior breast procedures occurred several decades earlier, highlighting that foreign body reactions may present long after the original intervention and may be difficult to recognize when historical procedural details are limited.

Although foreign body granulomatous reactions of the breast have been previously reported, they remain an important and potentially misleading mimic of invasive breast carcinoma on imaging. In the present case, imaging demonstrated features highly suspicious for malignancy, including architectural distortion on mammography and an irregular hypoechoic mass with posterior acoustic shadowing on ultrasound. However, histologic evaluation revealed foreign body granulomatous inflammation without evidence of malignancy. These histologic findings were considered radiologic-pathologic concordant because the biopsy targeted the imaging abnormality, and the benign granulomatous process adequately explained the suspicious imaging features. Careful radiologic-pathologic correlation, therefore, supported a benign diagnosis, allowing avoidance of unnecessary surgical excision and supporting conservative management with imaging surveillance. Radiologic-pathologic concordance is an essential step in the evaluation of breast biopsies and guides appropriate clinical management following image-guided sampling [4,5].

This case underscores the importance of recognizing benign post-procedural changes and foreign body reactions as potential mimics of malignancy on breast imaging. In patients with a history of prior breast interventions, these lesions may present as suspicious masses and lead to biopsy. Awareness of this entity and careful radiologic-pathologic correlation are critical to establishing diagnostic concordance, guiding appropriate management, and preventing unnecessary surgical intervention. The patient remained clinically stable on follow-up, and continued imaging surveillance was recommended to ensure stability of the lesion.

## Conclusions

Foreign body granulomatous reactions of the breast represent an important benign mimic of malignancy on breast imaging. As demonstrated in this case, such lesions may present with imaging findings highly suspicious for carcinoma, including architectural distortion and irregular hypoechoic masses with posterior acoustic shadowing. Recognition of benign imaging mimics and careful radiologic-pathologic correlation are essential to establish concordance following biopsy and guide appropriate management. Multidisciplinary collaboration between radiologists and pathologists is important in avoiding unnecessary surgical intervention when benign pathology adequately explains suspicious imaging findings.
